# Impacts of diet supplemented with antioxidants (vitamin E and selenium) and condensed tannins on the growth performance, carcass, blood biochemistry, and hematological traits in growing rabbits

**DOI:** 10.5455/javar.2025.l937

**Published:** 2025-08-18

**Authors:** Mahmoud Kamal, Ahmed K. Aldhalmi, Ahmed I. Elsherbeni, Islam M. Youssef, Mahmoud I. S. Hassan, Yanfen Cheng, Garsa Alshehry, Mohamed E. Abd El-Hack

**Affiliations:** 1 Laboratory of Gastrointestinal Microbiology, National Center for International Research on Animal Gut Nutrition, Nanjing Agricultural University, Nanjing, China; 2 Animal Production Research Institute, Agricultural Research Center, Giza, Egypt; 3 College of Pharmacy, Al-Mustaqbal University, Babylon, Iraq; 4 Animal Production Department, Faculty of Agriculture, Al-Azhar University, Assiute, Egypt; 5 Department of Food Science and Nutrition, College of Sciences, Taif University, Taif, Saudi Arabia; 6 Poultry Department, Faculty of Agriculture, Zagazig University, Zagazig, Egypt.; 7 Department of Industrial Pharmacy, College of Pharmaceutical Sciences and Drug Manufacturing, Misr University for Science and Technology (MUST), Giza, Egypt

**Keywords:** Condensed tannins, growth performance, rabbit, selenium, summer, vitamin E

## Abstract

**Objective:**

This study aimed to investigate the effect of antioxidants in the diet, vitamin E (V.E.), selenium (Se), and condensed tannins (COT), on growth performance, carcass characteristics, and certain physiological parameters of weaner rabbits.

**Materials and Methods:**

In a completely randomized design experiment, 105 weaning New Zealand White rabbits were reared from August to October (31.5°C ± 2°C) and fed a basal diet *ad libitum* for 8 weeks. They were then randomly assigned to seven nutritional treatments. As the control group, a basic diet devoid of antioxidants was used, while the other six groups were fed diets containing 100 or 200 mg V.E./kg, 0.1 or 0.2 mg Se/kg, and 1.5 or 3.0 gm COT/kg.

**Results:**

The findings showed that dietary interventions had no discernible impact on weight gain. The control group’s feed consumption (FC) was substantially higher than other treatments. In comparison to the control, the feed conversion ratio (FCR) increased (*p *< 0.05) with 0.1 mg Se/kg diet (23.71%), V.E. 200 mg/kg diet (20.21%), 0.2 mg Se/kg diet (20.21%), and 1.5 gm COT/kg diet (15.27%). None of the dietary supplements under investigation had a discernible impact on the rabbits’ carcass characteristics or blood serum metabolites, except alanine aminotransferase (AST) and white blood cells (WBCs). While AST was enhanced after receiving COT 1.5 or 3.0 gm, Se 0.2 mg, and V.E. 200 mg/kg diet by 37.0%, 29.6%, 29.2%, and 29.0%, respectively, WBCs were reduced after receiving V.E. 200 mg/kg, Se 0.2 mg, and COT 3.0 gm/kg diet by 56.2%, 41.4%, and 29.13%, respectively.

**Conclusion:**

During the summer, feeding rabbits extra (V.E. 200 mg, Se 0.1 and 0.2 mg, or COT 1.5 and 3.0 gm/kg diet) appeared to help with FCR and FC.

## Introduction

Heat stress (HS) is the greatest threat to animal production today [1]. It negatively affects animal physiology, behavior, and health, eventually harming animal reproduction and survivability [1,2]. Rabbits are considered the best meat producers because of their excellent growth rates, high meat quality, and feed efficacy [3]. Additionally, FAO [4] estimated that the amount of rabbit meat produced worldwide is 1,482,441 tons of equivalent carcasses. Given these circumstances, there is growing concern about growing the rabbit industry as a cost-effective and ecologically sound animal farming system [5]. In this sense, HS significantly affects the physiological aspects of rabbit production, particularly in tropical or subtropical regions [6,7]. Additionally, Abdelnour et al. [8] stated that rabbits are thermoneutral between 15°C and 25°C, with increased temperatures leading to stress and/or discomfort.

Weaned rabbits demonstrate an increased susceptibility to HS because of psychological shock, physiological changes, respiratory issues, exhaustion, elevated cardiovascular activity, anorexia, and a drop-in basic metabolic rate [9,10]. As per numerous reports, HS has been linked to adverse effects on growth, effectiveness of feed, and meat quality [5,8,11]. Furthermore, by weakening the immune system, altering the redox state, and causing inflammatory responses, HS may compromise the welfare and health of growing rabbits [12]. Developing effective management practices for rabbit growth is crucial to improve well-being and meat quality, reducing the negative impact of HS on rabbit growth and optimizing the rabbit industry’s economics [13].

Serra et al. [14] found that dietary antioxidants protect rabbit tissues from oxidative damage, but research on selenium (Se) or vitamin E (V.E.)’s impact on rabbit growth performance has yielded different results. According to Trombetti et al. [15], the addition of V.E. and Economas ETM to rabbit diets acts as an antioxidant, increasing oxidation resistance and improving product stability by reducing degradation processes. Additionally, Amer et al. [16] indicated that adding Se to rabbit feed improves meat quality and rabbit growth performance. Viliene et al. [17] found that supplementation with organic Se and V.E. had no negative effects on growth while significantly improving the quality of rabbit muscle meat.

According to Huang et al. [18], there are three primary classifications of tannins: phlorotannins, condensed tannins (COT), and hydrolysable tannins. A complicated class of polyphenolic substances is known as tannins. As stated by Sinaga et al. [19], tannin from 0.25% chestnut extract could replace growth promoters and antibiotics in rabbit rations, enhancing gut health without impacting growth. Mancini et al. [20] suggest tannin as a potential nutritional supplement. According to Liu et al. [21], the natural chestnut wood extract Silvafeed ENC had pro- and positive-oxidant effects at 0.5% and 1% on rabbit carcass and meat attributes without any negative effects. This research investigated the effects of V.E., Se, and COT on the growth efficiency, carcass characteristics, and blood physiology of New Zealand white rabbits (NZWs) raised during the summer.

## Materials and Methods

### Ethical approval

The Institutional Animal Care and Use Committee’s rules were followed when conducting the experiments. They were approved by the Animal Production Research’s Institutional Ethics Committee at the Institute, Agricultural Research Center, Dokki, Giza 12618, Egypt.

### Animals and management

The current study was carried out at a private rabbitry farm in El Kassassin City, Ismailia Governorate, Egypt. This study employed 105 NZW rabbits in good health and free of external parasites, distributed into seven experimental treatments, 15 rabbits per treatment. The animals weighed 686 ± 13 gm at 5 weeks of age. Each animal was kept in a cage measuring 50 × 50 × 40 cm, which was wired and galvanized.

Water and feed were provided. During the experiment, all animals were kept under identical environmental circumstances, and light/dark cycles were maintained at 16 h of illumination and 8 h of darkness. The baseline diets were formulated to meet the nutritional requirements outlined by the NRC [22] for growing rabbits (Table 1). Agrivet, Egypt, provided V.E. through α-tocopherol acetate and Se in sodium selenite. Silvateam, Italy, supplied COT in the form of Silvafeed^®^ ATX, which is composed of 85% polyphenols, including vescalagin, castalagin, roburin, procyanidins, proanthocyanidins, catechins, epigallocatechins, quercetin, and others. V.E. or Se levels were considered when adjusting vitamin–mineral premixes.

### Experimental design

Each animal was weighed separately, and 7 groups for experimentation, each of 15 rabbits, were chosen at random. As a control group, the 1st group received only the basal diet. The other six groups of experiments were given the base diet plus 100 or 200 mg of V.E. per kg, 0.1 or 0.2 mg of Se per kg, or 1.5 or 3.0 gm of COT per kg of diet. It took eight weeks to complete the feeding trial. From August to October of 2022, the dietary trial continued for 8 weeks.

### Measurements

**Growth performance**

Rabbit’s weight gain was calculated once a week during the experimental period. It was calculated by subtracting its live body weight (LBW) at the beginning of each experimental week from its LBW at the end of the same week. The variation between the feed provided each week and the remaining amount after each week was utilized to calculate each rabbit’s mean weekly feed consumption (FC). The kg of feed needed for producing 1 kg of total body weight gain (TBWG) was utilized to estimate the FCR. The percent of change between each treatment is calculated compared to the control to show the differences between the treatments. Therefore, the control set is represented by the value zero. It is calculated using the following method:


$$\mathrm{Percent}\;\mathrm{of}\;\mathrm{change}\;=\;\frac{(\mathrm{Control}\;g\mathrm{roup}\;–\;\mathrm{Treatment}\;\mathrm{group})}{(\mathrm{Contro}l\;g\mathrm{roup})}\;\times \;100$$

Throughout the trial, the rabbits’ mortality was monitored and recorded daily. The overall mortality rate was ascertained by estimating the difference between the number of dead rabbits and the number of rabbits at the start of the experiment.

**Table 1.** Ingredients and calculated chemical composition of the basal diet.

Ingredients	(%)
Alfalfa hay	26.5
Yellow corn	15.0
Barley	17.0
Soybean meal (44%)	16.0
Wheat bran	20.0
Alfalfa straw	3.0
Limestone	1.65
Premix *	0.30
NaCl	0.30
Di- methionine	0.1
Anti-toxin	0.1
Anti-coccidia	0.05
Total	100.0
Chemical composition (as DM basics).	
CP	17.5
Fat	2.8
Digestible energy (kcal/kg)	2600
CF	10.0
Calcium	0.93
Lysine	0.84
Total phosphorus	0.62
Methionine	0.65
Methionine + Cysteine	0.63

*Supplied per 1 kg diet: 6000 IU vit. A; 900 IU vit. D3; 40 mg vit. E; 2.0 mg vit. K3; 2.0 mg vit. B1; 4.0 mg vit. B2; 2.0 mg vit. B6; 0.010 mg vit. B12; 5.0 mg vit. PP; 10.0 mg vit. B5; 0.05 mg B8; 3.0 mg B9; 250 mg choline; 50.0 mg Fe; 50.0 mg Zn; 8.5 mg Mn; 5.0 mg Cu; 0.20 mg I, and 0.00 mg Se; CP, crude protein; CF, crude fiber.

**Carcass traits**

After the 8-week experiment, five rabbits were randomly selected from each treatment and slaughtered to collect blood samples and carcass measure traits. Preparing for slaughter, the chosen animals’ body weights were measured the following morning after they had fasted for 12 h. The animals were fully exsanguinated after being beheaded. The intestines were eliminated and separated, the abdomens were cracked open, and the feet and tail were exposed after their bodies were chopped. Kamal et al. [23] stated that each empty carcass (devoid of the head, heart, lung, kidney, and liver) was measured, and a protocol was established to record the fur, head, and intestines. 

**Blood sampling**

To minimize the potentially confusing impact of varying blood chemistry throughout the day, the rabbits were slaughtered between 7:00 and 10:00 am. Every animal’s blood was drawn into two different pipes. White blood cells (WBCs) were measured in fresh blood samples using NIHON KOHDEN equipment (automated hematology analyzer). An initial specimen was taken, placed in a tube that had been heparinized, and examined for hematological indicators (red blood cells, hemoglobin, hematocrit, and platelets). The additional sample was taken in a non-heparinized tube to separate the serum and was centrifuged for 15 min at 3,000 rpm.

Before the serum biochemical parameters were analyzed, the serum specimens were preserved at (–20°C). The blood serum’s levels of triglycerides, total cholesterol, low-density lipoprotein (LDL), and high-density lipoprotein (HDL), in addition to the liver activity of the enzymes of ALT and AST, as per Young and Friedman [24], were measured calorimetrically utilizing commercial kits (Reactivos GPL CHEMELEX, S.A. Pol. Ind. Can Castells. C/Industria 113, Nau J 08420 Canovelles–Barcelona).

**Economic efficiency (EE)**

Initially, we multiplied the quantity of FC throughout the trial by the cost of 1 kg of each testing diet (calculated using the local prices in effect at the time of the test). Next, the net revenue (price per kilogram of rabbit) was calculated by deducting the aggregate feed cost from the overall revenue. According to Kamal et al. [23], the EE was computed by splitting net revenue by overall feed expenditure. The expenses for housing, labor, veterinary care, and rabbit purchases were not included because these costs were identical for all treatments.

### Examining data statistically

The data were analyzed using the SPSS General Linear Model procedure [25]. Duncan’s multiple-range test [26] and the one-way ANOVA test were used to evaluate mean differences. The following model was used:

*Y_ij_* = µ + *T_i_* + *E_ij_*;

where *Y_ij_*, individual observation; µ, overall mean; *T_i_*, effect of treatment (*i* = 1…7); and *E_ij_*, experimental error.

## Results

### Growth efficiency and mortality rate

Throughout the experiment, average rabbit temperatures were 31.5°C ± 2°C, and average daytime relative humidity was 57%. The impacts of antioxidants’ addition on final body weight, body weight gain, FC, feed conversion ratio (FCR), and mortality rate are shown in Table 2. The average BW of the 5-week-old rabbits in each treatment group was 686 ± 13 gm (*p *≥ 0.05), suggesting a fully randomized allocation of animals into the tested groups. The results showed that V.E., Se, and COT enhanced (*p *≤ 0.05) the FC and FCR compared to the control. The outcomes of the ANOVA revealed no substantial variations (*p *≥ 0.05) because of the treatments in FBW and TBWG. Nonetheless, notable changes were observed in FC and FCR among the trial groups due to the therapies.

**Figure 1.**
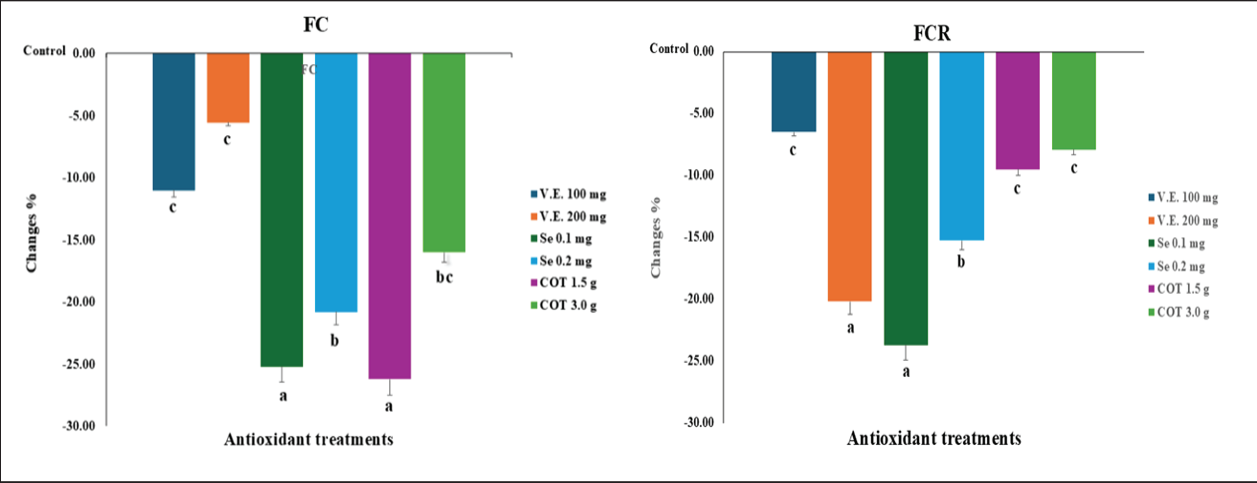
Changes in percent in FC and feed conversion ratio of growing rabbits between the control and treatment groups.

To show the differences between the treatments, the percentage of change between each treatment is calculated compared to the control. The control group was used as a starting point. The rabbit groups that were fed diets with Se 0.1 mg, V.E. 200 mg, and Se 0.2 mg/kg of diet increased FCR by 23.71%, 20.21%, and 15.27%, respectively. The diets with COT 1.5 gm, Se 0.1, and 0.2 mg/kg of diet increased FC by 26.21%, 25.2%, and 20.8%, respectively. The Se 0.1 and 0.2 groups showed the greatest overall improvements in FC and FCR (Fig. 1).

### Carcass characteristics

Table 3 illustrates the impacts of antioxidant treatments on various carcass attributes, including giblets, the total edible part, dressing yield, pre-slaughter weight, empty carcass weight, and abdominal fat weight. The analysis of variance revealed no statistically significant differences (*p *≥ 0.05) between treatments for any of the carcass traits.

### Blood parameters

Table 4 shows the impact of the treatments on the hematological and biochemical characteristics of blood. Significant variations in AST and WBCs were found between treatments using analysis of variance. On the other hand, most hematological characteristics and the remaining blood biochemical indicators revealed no statistically significant variations (*p *≥ 0.05) between treatments. The levels of AST were significantly decreased (*p *≤ 0.05) in growing rabbits fed 3.0 gm/kg COT, 200 mg/kg V.E., and 0.2 gm/kg Se than in rabbits fed other treatments. Similarly, the animals given 200 mg/kg V.E., 0.2 gm/kg Se, and 3.0 gm/kg COT had the lowest WBC levels (*p *≤ 0.05). This indicates that the use of antioxidants in various experimental groups significantly enhanced the health of rabbits.

**Figure 2.**
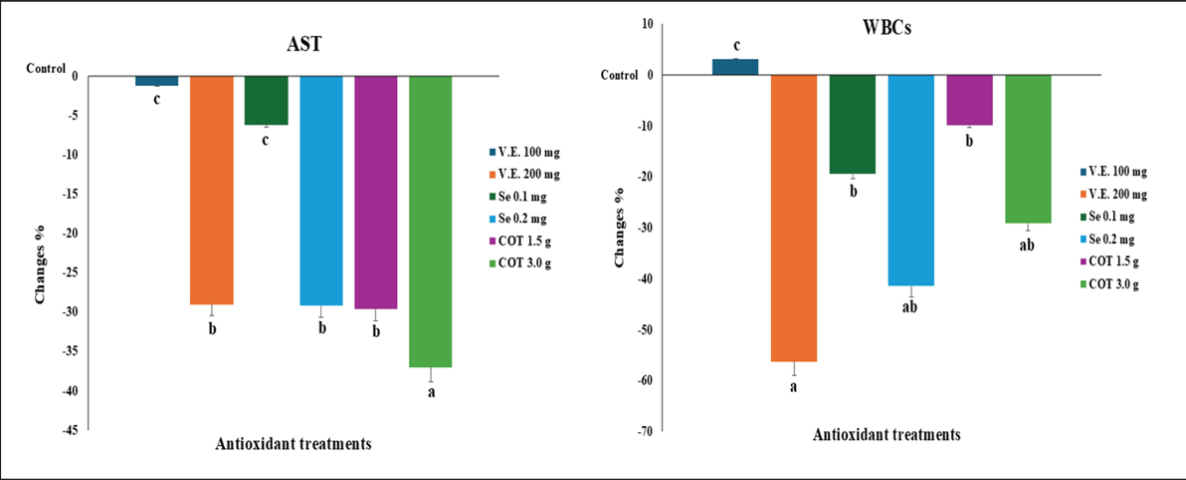
Changes in percent in AST and WBCs of growing rabbits between the control and treatment groups.

The AST was decreased by 37.0%, 29.6%, 29.2%, and 29.0%, respectively, after receiving antioxidant treatments at COT 3.0, 1.5 gm, Se 0.2 mg, and V.E. 200 mg/kg diet. Additionally, compared to the other groups, the WBCs were decreased by 56.2%, 41.4%, and 29.13%, respectively, by the antioxidant treatments of V.E. 200, Se 0.2 mg, and COT 3.0 gm/kg diet. Compared to the other groups, the AST and WBC levels in the antioxidant treatment groups (COT 3.0 gm, Se 0.2 mg, and V.E. 200 mg) were significantly higher (Fig. 2).

### Economic efficiency

**Figure 3.**
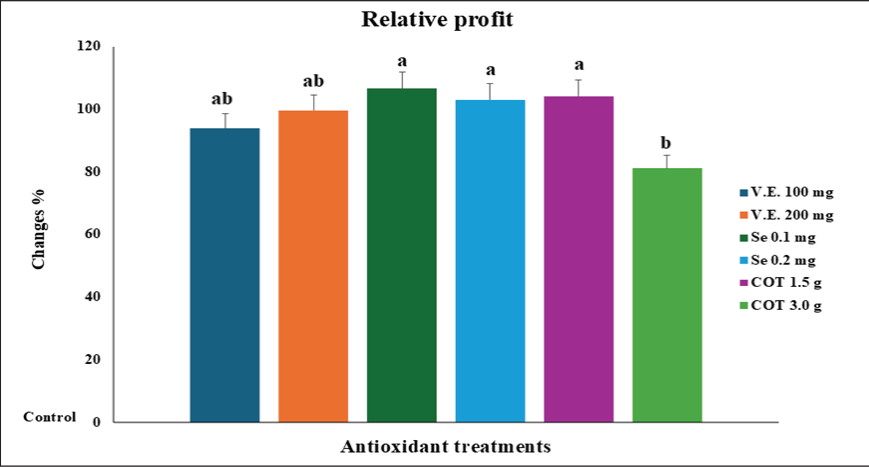
Changes in percent of relative profit between control and treatment groups.

Table 5 displays the various treatments’ EE. Most antioxidant treatments in this study produced a greater relative profit and EE than the control group. Figure 3 illustrates how antioxidant treatments of the diets containing Se 0.2 mg, COT 1.5 gm, and Se 0.1 mg/kg raised relative profits by 106.56%, 104.13%, and 102.95%, respectively, compared to nutrition under control (100%).

**Table 2.** Effects of dietary antioxidant and condensed tannins supplementation on the performance of growing rabbits (Means ± SE).

Traits	Treatments (antioxidant)							*p*-value
	Control	Vitamin E		Se		Condensed tannins		
		100 mg	200 mg	0.1 mg	0.2 mg	1.5 gm	3.0 gm	
IBW (gm)	687.5 ± 36.6	687.3 ± 37.2	687.6 ± 40.2	687.1 ± 34.7	686.1 ± 30.7	685.9 ± 30.6	685.3 ± 36.2	0.982
FBW (gm)	1859.2 ± 48	1683.2 ± 12	1672.8 ± 12	1709.3 ± 14	1782.5 ± 46	1704.1 ± 13	1524.7 ± 16	0.598
TBWG (gm)	1171.7 ± 25	1049.7 ± 82	1028.5 ± 74	1077.6 ± 80	1096.4 ± 30	1066.1 ± 81	918.8 ± 98	0.352
FC (gm)	5060.0^a^ ± 43	4203.3^b^ ± 30	3536.7^c^ ± 25	3540.0^c^ ± 25	4000.0^b^ ± 80	3496.7^c^ ± 25	3680.0^c^ ± 38	0.000
FCR	4.34^a ± ^0.08	3.76^ab^ ± 0.27	3.21^b^ ± 0.23	3.07^b ± ^0.23	3.66^ab ± ^0.81	3.48^b ± ^0.36	3.48^bc ± ^0.36	0.002
Livability	1.00 ± 00	0.93 ± 067	0.93 ± 067	0.93 ± 067	0.93 ± 067	0.93 ± 067	0.87 ± 091	0.914

^a, b, c ^Means within the same row with different superscripts are significantly different (*p *≤ 0. 05).FBW, final body weight; FC, feed consumption; FCR, feed conversion ratio; IBW, initial body weight; TBWG, total body weight gain.

**Table 3.** Effects of dietary antioxidants and condensed tannins supplementation on the carcass traits of growing rabbits (Means ± SE).

Traits	Treatments (antioxidant)							*p* - value
	Control	Vitamin E		Se		Condensed tannins		
		100 mg	200 mg	0.1 mg	0.2 mg	1.5 gm	3.0 gm	
Pre-slaughter weight (gm)	1912.8 ± 79.8	1850.0 ± 41.8	1796.0 ± 58.5	1759.2 ± 55.5	1707.2 ± 34.9	1804.4 ± 29.2	1773.2 ± 62.4	0.219
Empty carcass (gm)	1052.0 ± 51.5	1023.6 ± 23.3	982.8 ± 39.7	961.2 ± 23.4	926.4 ± 34.2	957.6 ± 51.5	931.6 ± 32.5	0.217
Dressing yield (%)	54.98 ± 1.3	55.33 ± 0.5	54.68 ± 0.8	54.71 ± 0.9	54.22 ± 1.3	53.02 ± 2.5	52.55 ± 0.6	0.712
Giblets (gm)	78.8 ± 4.27	71.2 ± 6.56	64.4 ± 3.71	64.4 ± 2.64	69.2 ± 3.20	68.4 ± 4.26	63.6 ± 4.44	0.203
Edible parts (%)	59.10 ± 1.3	59.16 ± 0.5	58.29 ± 0.8	58.38 ± 1.1	58.26 ± 1.39	56.80 ± 2.6	56.17 ± 0.86	0.681
Abdominal fat (gm)	15.6 ± 3.2	12.8 ± 2.4	14.8 ± 2.2	11.2 ± 1.1	12.8 ± 2.3	12.0 ± 3.8	10.4 ± 1.9	0.780

## Discussion

The findings of the current study indicate that while there were no noteworthy differences in the FBW and TBWG, there were notable differences in the FC and FCR treatments among the experimental groups. These outcomes align with Ebeid et al. [27], who indicated that while FCR lowered in the rabbits, supplementary V.E., natural Se, and V.E.+ Se raised the FBW and daily gain. According to Ragab et al. [28], V.E. is given orally to rabbits as an antioxidant that helps to maintain growth performance and digestion.

Additionally, Minardi et al. [29] found that dietary supplements containing Se (0.15 or 0.30 mg/kg diet) or V.E. (100 or 200 mg/kg diet) had no impact on the performance of growth. The results indicate that the best FCR and the lowest amount of FC were in the Se and V.E. groups. This finding may be due to the importance of Se and V.E. as food supplements for rabbits. Se, an essential trace element with antiviral and antibacterial properties, greatly improves cell integrity and production efficiency. On the other hand, rabbit diets contain either very little or no Se, which can negatively affect the health of rabbits. The rationale may be that Se exhibits high absorption, enabling it to traverse the intestinal membrane and enter the bloodstream via active transport. Se facilitates normal cellular growth and plays a crucial role in regulating transcription factors and cellular signaling pathways [30]. Additionally, Se enhances the metabolism of thyroid hormones, which is vital for normal growth and metabolic processes, as selenoenzymes modulate various stages of thyroid hormone metabolism [31].

Viliene et al. [17] stated that growing rabbits can benefit from using both Se forms and additional V.E. without experiencing any negative growth-related side effects. Additionally, Hassan et al. [32] state that organic Se sources such as yeast or algae can be used in growing rabbit diets without causing any adverse effects on growth performance. Additionally, they have beneficial effects in improving the antioxidative status. According to Mohamed et al. [33], broiler chicks under HS may benefit from adding 100 mg Zn + 1.5 mg Cr + 0.6 or 0.9 mg Se from yeast, alone or in combination. On the other hand, Abd El-Hack et al. [34,35] reported that broiler productivity increased when Se nanoparticles were added to the diet; however, decreased Se levels (1.5 ml/kg diet) demonstrated favorable findings.

**Table 4.** Effects of dietary antioxidant and condensed tannins supplementation on the blood biochemical and hematological traits of growing rabbits (Means ± SE).

Traits	Treatments (antioxidant)									*p* - value
	Control	Vitamin E			Se			Condensed tannins		
		100 mg	200 mg	0.1 mg		0.2 mg	1.5 gm		3.0 gm	
TC (mg/dl)	74.0 ± 5.6	66.8 ± 7.3	75.4 ± 9.6	93.0 ± 13.2		67.6 ± 11.5	75.6 ± 8.2		84.6 ± 10.6	0.503
TG (mg/dl)	71.2 ± 9.1	86.6 ± 8.8	76.4 ± 11.8	79.2 ± 6.3		89.8 ± 5.3	73.0 ± 5.2		82.6 ± 10.3	0.680
HDL (mg/dl)	29.8 ± 3.9	24.6 ± 1.3	29.2 ± 2.9	32.6 ± 3.4		27.0 ± 2.9	24.8 ± 1.5		34.0 ± 4.3	0.257
LDL (mg/dl)	29.8 ± 7.3	25.0 ± 5.6	30.8 ± 10.8	44.4 ± 11.8		22.6 ± 9.2	36.4 ± 6.7		34.0 ± 8.6	0.645
ALT (U/l)	60.2 ± 6.7	53.2 ± 8.6	42.2 ± 4.5	59.8 ± 8.34		55.8 ± 8.8	65.2 ± 5.2		50.6 ± 6.0	0.369
AST (U/l)	70.4^a^ ± 7.3	67.8^a^ ± 5.3	48.0^bc^ ± 2.7	61.6^ab^ ± 6.5		49.2^bc^ ± 4.6	58.6^bc^ ± 4.5		42.6^c^ ± 4.8	0.006
WBCs (10^3^/mm^3^)	7.84^a^ ± 0.8	7.6^a^ ± 1.1	3.2^c^ ± 1.4	6.3^ab^ ± 0.9		4.3^bc^ ± 0.5	6.6^ab^ ± 1.4		5.0^bc^ ± 0.4	0.014
RBCs (10^6^/mm^3^)	6.41 ± 1.31	5.32 ± 0.43	5.38 ± 1.13	5.86 ± 0.73		5.72 ± 1.47	6.12 ± 0.88		5.46 ± 0.52	0.362
Hb (gm/100 ml)	11.57 ± 0.4	11.81 ± 0.7	11.56 ± 0.6	11.81 ± 0.2		11.62 ± 0.3	11.80 ± 0.1		11.67 ± 0.7	0.986
Hematocrit (%)	38.64 ± 3.2	39.14 ± 1.2	39.07 ± 1.7	38.82 ± 2.5		38.96 ± 1.8	38.84 ± 2.7		38.95 ± 1.1	0.975
Plt (×10^3^/mm^3^)	279 ± 72.6	264 ± 60.8	310 ± 12.4	276 ± 82.6		251 ± 92.3	271 ± 82.6		286 ± 72.1	0.284

^a, b, c ^Means within the same row with different superscripts are significantly different (*p *≤ 0. 05).ALT, alanine aminotransferase; AST, aspartate aminotransferase; Hb, hemoglobin; HDL, high-density lipoprotein; LDL, low-density lipoprotein; Plt, platelets; RBCs, red blood cells; TC, total cholesterol; TG, triglycerides; WBC, white blood cell.

According to Mancini et al. [20], adding tannins to rabbit feed does not hurt productivity or digestibility. It only slightly raises the antioxidant level so that it can be used as a feed additive. Likewise, according to Sinaga et al. [19], tannin obtained from a 0.25% chestnut extract can be considered a feasible addition to rabbit rations during the growing season. It may replace growth promoters and antibiotics in rabbit farming and improve gut health without impacting the rabbit’s growth. In contrast, increasing antioxidant supplementation had no discernible impact on improving rabbits’ growth efficiency [36,37].

**Table 5.** Input/output analysis and EE of trial groups.

Traits	Treatments (antioxidant)						
	Control	Vitamin E		Se		Condensed tannins	
		100 mg	200 mg	0.1 mg	0.2 mg	1.5 gm	3.0 gm
Total body weight gain (gm)	1171	1049	1028	1077	1096	1066	918
Total feed consumption (kg)	5.060	4.203	3.536	3.540	4.000	3.496	3.680
Feed cost (LE)*	32.89	27.39	23.10	23.09	26.19	23.51	24.47
Gain price (LE) **	81.97	73.43	71.96	75.39	76.72	74.62	64.26
Profit above feed cost***	49.08	46.04	48.86	52.3	50.53	51.11	39.79
Relative profit (%) ****	100	93.80	99.55	106.56	102.95	104.13	81.07

* = total feed consumption × price (price of 1 kg control diet was 6.5 L.E., price 2022).One kg of V.E. costs 170 L.E. The price of 1 kg of diet plus 100 mg and 200 mg V.E. was 6.517 and 6.534 LE, respectively. One kg of Se costs 2400 LE. The price of one kg of diet plus 0.1 mg and 0.2 mg of Se was 6.524 and 6.548 L.E., respectively. One kg of COT costs 150 L.E. The price of one kg of diet plus 1.5 or 3.0 gm of COT was 6.725 and 6.950 L.E., respectively.** = total gain × 70 (one kg 70 L.E).*** = gain price – feed cost**** = relative profit for treatment/ net revenue of control × 100.

According to our findings, none of the carcass attributes showed statistically significant differences between antioxidant treatments. Similarly, Selim et al. [38] showed that the vitamin treatments had no discernible effect on the weight of the pre-slaughter carcass, dressing %, or spleen. Furthermore, Mohamed et al. [39] indicated that the carcass traits were not affected by Se supplementation in rabbit diets. Furthermore, Dalle Zotte et al. [40] indicated that there was no discernible difference in the weight of carcass traits between rabbits treated with vitamins and the control groups.

These results align with the results of Minardi et al. [29], who discovered that dietary supplements of V.E. and Se did not impact rabbit meat’s carcass or meat characteristics, demonstrating that the meat had good nutritional value. According to Mohamed et al. [33], when broiler chicks are stressed by heat, adding yeast can improve the carcass’s features; however, the best dosage of organic Se with a zinc–chromium mixture has no discernible effects on health or efficiency. According to Sinaga et al. [19], adding tannins from chestnut extract to rabbit rations can have a major effect on the percentage of meat, bone, and fat, as well as the percentage of carcasses. This could mean that natural antibiotics are replaced during the fattening process. Additionally, Mancini et al. [20] observed no appreciable variations in the growth, carcass characteristics, or digestibility of rabbits fed a mix of tannins. Conversely, as an antioxidant, V.E. can greatly enhance the qualities of rabbit carcasses, particularly in areas where the nutrient is deficient. For this reason, V.E. is an important source of nutrition for Black Baladi rabbits that have been weaned [28].

Researchers employ blood biochemical indicators to assess the health and physiological status of animals [23,41]. The liver is crucial for digestive and metabolic functions and is susceptible to various degrees of chemical and biological damage. Elevated liver enzyme levels are unequivocally detrimental. Their amounts can alter bodily functions, adversely affecting health and diminishing production efficiency [42]. AST and ALT values in blood tests serve as indications of hepatic function and injury [43,44]. The body’s response to disease associates elevated levels of these enzymes with hepatic or muscle injury [45,46]. The obtained results showed that AST and WBC levels were lower in the treatments than in the control group. However, there were no other significant differences in the hematological or biochemical features of the blood. Goda et al. [47] also found that AST, ALT, triglycerides, and total cholesterol levels were significantly lower (*p *≤ 0.05) in rabbits that were fed V.E. compared to rabbits that were not fed it. According to Desoky [48], V.E. boosts the immune system and keeps leukocytes and macrophages safe during phagocytosis, making the body less likely to become sick. In this way, Adeyemo et al. [49] discovered that giving V.E. to rabbits did not have any negative effects on them. Supplementation with V.E. and Se did not adversely affect the blood parameters of NZW rabbits [50]. Additionally, Ragab et al. [28] suggest that V.E., an antioxidant, could be beneficial for rabbits living in hot regions. Supplementing rabbit diets with V.E. in these areas helps maintain animal health by regulating blood parameters and balancing oxidative activity.

Additionally, Hafth et al. [51] found that the hematological and biochemical indicators improved more (*p *< 0.05) in the treated V.E. rabbits than in the control. In addition, a rise in DL-α-tocopherol can decrease cholesterol and improve α-tocopherol accumulation in muscles, according to Vilienė et al. [52]. Giving yeast and Se to broiler chicks that were raised at high temperatures can make their blood biochemicals, antioxidant status, and hematological parameters better [33]. Moreover, Mancini et al. [20] observed that feeding rabbits a mix of tannins exhibited favorable effects on plasma enzyme concentration and improved health in rabbits. V.E. and Se have been shown to have a positive effect on WBCs. By getting rid of free radicals and strengthening the immune system, these nutrients may help increase the production of WBCs. In this study, WBCs dropped by 56.2% compared to the control group. This result shows that the nutritional supplements lowered HS, which in turn lowered the production of WBCs to fight infections caused by damaged cells.

The findings showed that most antioxidants and COT treatments in this study yielded higher relative profit and EE than the control. The current findings are compatible with those of Ragab et al. [28] and El-Moniem et al. [53], who found that compared to control rabbits, V.E. could increase total revenue, net revenue, EE, and EER more. Furthermore, it was noted by Abd Allah et al. [54] that V.E. supplementation increased revenue and decreased feed cost/kg for growing rabbits. In the same vein, Dalle Zotte et al. [40] stated that the greatest EE was observed when comparing rabbits fed a V.E.-free diet to those fed one with a V.E. supplement. Similarly, tocopherol and tocotrienol have both been shown to offer durability against oxidation, which is proven by the growth efficiency of animals and, as a result, increased profitability [55]. In addition, Abdel-Khalek et al. [50] suggested adding tannins or Se to the diet of rabbits during times of HS to help them utilize their feed efficiently and achieve greater EE.

## Conclusion

This study evaluated the effects of various antioxidant treatments on rabbits. The treatments included V.E. (100 and 200 mg), Se (0.1 and 0.2 mg), and COT (1.5 and 3.0 gm per kg of diet). The results showed that these antioxidants improved the feed conversion and FCR in rabbits. Additionally, they enhanced the levels of AST, increased WBCs, and improved overall profitability and EE compared to the control group. Overall, the study suggests that antioxidants, when used in conjunction with Se and COT, can promote growth, enhance rabbit performance, and improve their overall health.

## List of abbreviations

ALT, alanine aminotransferase; AST, aspartate aminotransferase; COT, condensed tannins; FBW, final body weight; FC, feed consumption; FCR, feed conversion ratio; gm, grams; Hb, hemoglobin; HDL, high-density lipoprotein; LBW, live body weight; LDL, low-density lipoprotein; NZW, New Zealand White rabbit; Plt, platelets; RBCs, red blood cells; Se, selenium; TBWG, total body weight gain; TC, total cholesterol; TG, triglycerides; V.E., vitamin E; WBCs, white blood cells.
